# A Conceptual Framework for Optimizing Blood Matching Strategies: Balancing Patient Complications Against Total Costs Incurred

**DOI:** 10.3389/fmed.2018.00199

**Published:** 2018-07-25

**Authors:** Joost H. J. van Sambeeck, Puck D. de Wit, Jessie Luken, Barbera Veldhuisen, Katja van den Hurk, Anne van Dongen, Maria M. W. Koopman, Marian G. J. van Kraaij, C. Ellen van der Schoot, Henk Schonewille, Wim L. A. M. de Kort, Mart P. Janssen

**Affiliations:** ^1^Department of Transfusion Technology Assessment, Sanquin Research, Amsterdam, Netherlands; ^2^Center for Healthcare Operations Improvement and Research, University of Twente, Enschede, Netherlands; ^3^Department of Donor Studies, Sanquin Research, Amsterdam, Netherlands; ^4^Sanquin Diagnostic Services, Amsterdam, Netherlands; ^5^Sanquin Research and Landsteiner Laboratory, Department of Experimental Immunohematology, Academic Medical Center, University of Amsterdam, Amsterdam, Netherlands; ^6^Department of Transfusion Medicine, Sanquin Blood Bank, Amsterdam, Netherlands; ^7^Department of Donor Affairs, Sanquin Blood Bank, Amsterdam, Netherlands; ^8^Department of Clinical Transfusion Research, Sanquin Research, Amsterdam, Netherlands; ^9^Department of Social Medicine, Academic Medical Center, Amsterdam, Netherlands

**Keywords:** blood supply chain, alloimmunization, cost-effectiveness, optimization, modeling

## Abstract

Alloimmunization is currently the most frequent adverse blood transfusion event. Whilst completely matched donor blood would nullify the alloimmunization risk, this is practically infeasible. Current matching strategies therefore aim at matching a limited number of blood groups only, and have evolved over time by systematically including matching strategies for those blood groups for which (serious) alloimmunization complications most frequently occurred. An optimal matching strategy for controlling the risk of alloimmunization however, would balance alloimmunization complications and costs within the entire blood supply chain, whilst fulfilling all practical requirements and limitations. In this article the outline of an integrated blood management model is described and various potential challenges and prospects foreseen with the development of such a model are discussed.

## 1. Introduction

**Figure 1:**
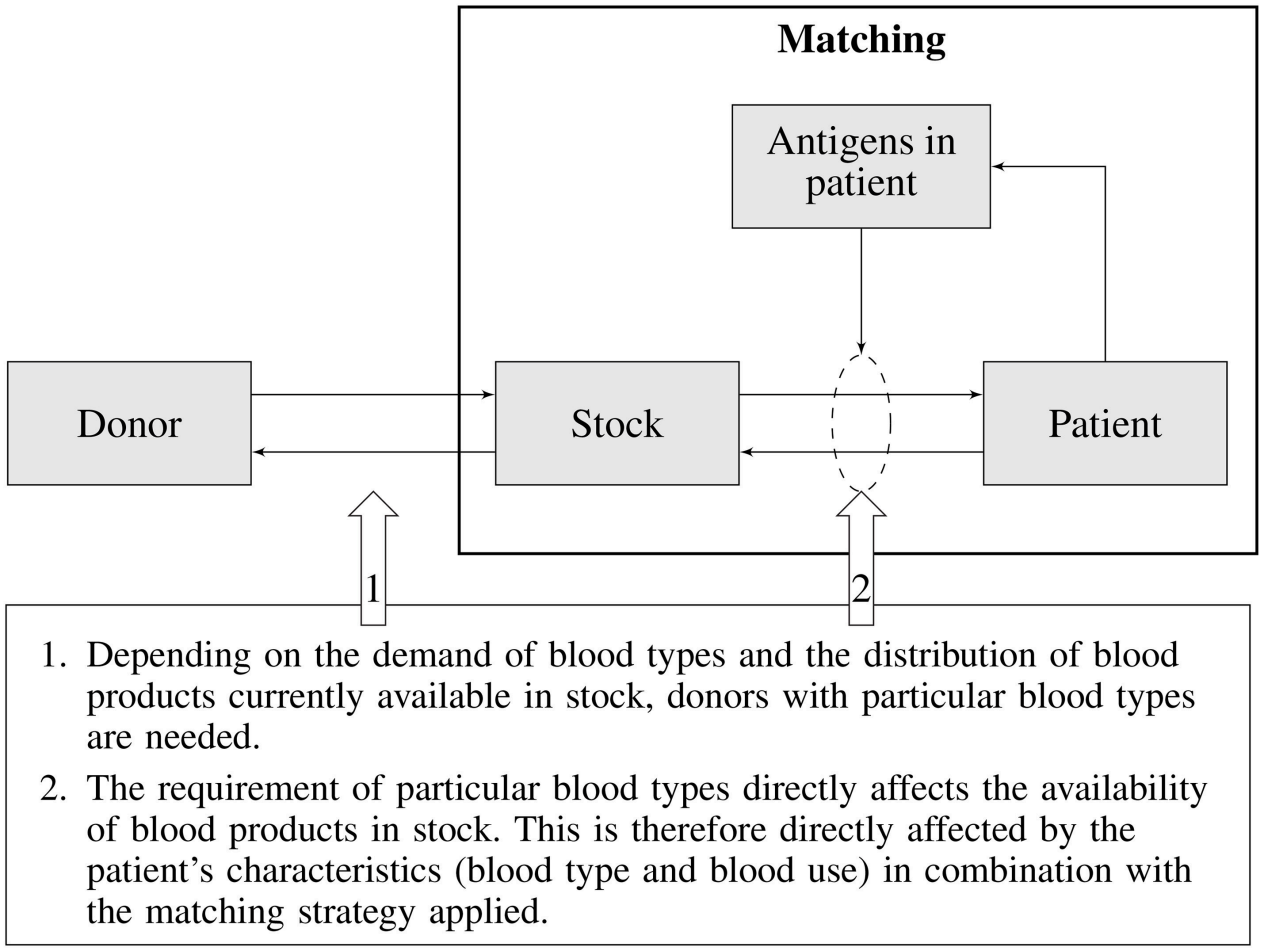
Schematic overview of blood type matching and its impact on the blood supply chain.

In a utopian world every blood transfusion would be handled like an organ transplant, which means that one would try to find a perfect match between donor and recipient. The reality however is that completely matched donor blood is impossible in practice due to the abundance of blood group antigens, costs associated with blood typing, and complications the logistics for such a scheme would impose. As a consequence only a handful of blood group antigens are matched, posing transfusion recipients at risk for alloimmunization and associated transfusion complications. An ideal matching strategy would be one that minimizes the risk of alloimmunization, is cost-effective, and fits within the practical limitations of the blood supply chain. In the past, matching strategies have been guided by the frequency of alloimmunization incidents, without systematically considering all consequences such strategies impose on the blood supply. Since a selected matching strategy will either directly or indirectly affect the entire blood supply chain (Figure 1), an integrated approach is required. Such an approach would, for any particular blood matching strategy, allow balancing the costs of donor recruitment, donor typing, inventory management, blood product logistics, patient blood typing, and alloimmunization complications in transfusion recipients. Besides costs also the effects of transfusion complications on patients health should be taken into account. This article describes the outline of a generic integrated blood management model, its components, their interaction and potential complicating factors and limitations currently foreseen for such a model.

We will first provide a description of all elements within the blood transfusion chain that are relevant to such a blood management model. Next we will describe how various elements are combined into an integrated model. Finally, we will discuss which challenges are foreseen with the implementation of the model and potential prospects. Challenges will concern knowledge required for shaping the modeling structure and the availability of data for various model parameters. Not only will the model guide the search for a rational choice of an optimal matching strategy, it will create transparency for the decision arena: the balance between costs and patient outcomes will become explicit for whatever optimal decision is selected. Secondly, by developing an integrated model, any blind spots in knowledge regarding any of the elements of the decision model will become visible and will have to be filled in.

The elements identified for the integrated blood management model are: the patient population, transfusion practice, pre-disposition of transfusion complications, typing and matching strategies, and the donor population. Note that as the patient is the primary concern, it is the patient that should be the starting point of the analysis. From there we will work our way back through the blood transfusion chain toward the donor population.

## 2. Transfused patients, exposure and transfusion complications

Blood transfusion is one of the most common medical procedures performed in hospitals. Despite its benefits, patients exposed to red blood cell (RBC) alloantigens may produce antibodies, which can cause acute or delayed hemolytic transfusion reactions (HTR). In addition, upon pregnancy in alloimmunized women, hemolytic disease of the fetus and new-born (HDFN) may occur. Not all patients form antibodies after RBC transfusion. According to current views, most are so-called non-responders and will never form antibodies despite numerous transfusions. Others seem to have an increased immunization risk and develop multiple antibodies after a few antigenic exposures, these are referred to as the (hyper) responders (1). It is currently not possible to prospectively identify patients that will form antibodies. In the absence of phenotypic matching, RBC alloimmunization risks vary between patient groups; it occurs in less than 5% of all transfusion recipients, increases to about 10–30% in patients with thalassemia, auto-immune hemolytic anemia or myelodysplastic syndromes, and can be more than 50% in sickle cell anemia patients (2, 3). In addition, patients with antibodies are at increased risk for additional antibody development upon subsequent transfusions (4, 5). During pregnancy, maternal RBC antibodies against paternal inherited antigens can pose the child at risk for HDFN. Besides anti-D, anti-E, anti-K, and anti-c are the most frequently encountered antibodies with the potential to seriously complicate pregnancy if the fetus carries the cognate antigen. The risk for severe HDFN in these fetuses, requiring intra-uterine or postnatal (exchange) transfusion, is estimated to be 12% for anti-K, 8.5% for anti-c and about 1% for anti-E. While for anti-D, administration of anti-D immunoglobulin (besides preventive D-matching) has reduced the risk of D immunization from 15% to 0.3%, such measures are not available or not always applied for other antigens, which are in the majority of cases elicited by previous transfusions (6).

The impact of transfusion reactions may vary widely, ranging from serologic observations or mild symptomatic anemia only, to life-threatening complications and death. It is obvious that with increasing severity, costs of treatment will also increase, although studies reporting on such associations and associated costs are currently limited or completely lacking (7). Maximum benefits of alloimmunization prevention can be obtained by administering extended antigen matched blood to patients who have an a priori high risk for alloimmunization. Therefore, unraveling genetic and environmental conditions enhancing RBC immunization would support preventive strategies. Although most studies on this subject have been performed in sickle cell disease (SCD) patients, factors such as age, sex, inflammatory status, MHC class-II genotype, polymorphisms associated with immune modulation and altered immune (regulatory) cells and disease or therapy associated immunosuppression seem to influence the immune response toward transfusion exposed alloantigens (1, 8–13). Due to logistic constraints, elaborate preventive matching based on a responder-profile is expected to be only feasible for a small proportion of patients. Targeting patients with (chronic) elective transfusions is likely to be feasible. Also, two recent prospective studies showed that less than 50% of surgery patients, who according to the local hospital pre-operative blood-ordering schedule had a high transfusion risk, were actually transfused. Extensive preventive matching as a routine policy is therefore expected to require a substantial amount of additional work and costs. Moreover, about 25% of patients required more than the anticipated number of RBC units during surgery and extended matched units were not readily available (14, 15).

As the blood management model is aiming to optimize strategies for preventing HTRs, the risk of alloimmunization in patients, its associated cost and health impact needs to be explicated. The ongoing Dutch R-fact study in which the predisposition for formation of antibodies is studied will allow modeling the likelihood of antibody formation. This information, combined with data on blood use for various patient groups, which will be obtained from the Dutch PROTON study (in which detailed transfusion data from a large number of hospitals are combined in a Dutch Transfusion Datawarehouse), will provide the information required to model the likelihood of HTRs in various patient groups. Research on the cost and health impact associated with HTRs will also be required to complete the model for patient and health outcome of transfusion complications.

## 3. Current matching strategies in the Netherlands

In the Netherlands all RBC transfusions are compatible for ABO and D antigens. Since 2011 the guideline for selection of RBC units prescribes preventive matching for specific blood group antigens for different patient subgroups. Since 2004 it has been policy to select K-negative RBCs for women aged under 45, which in 2011 was extended with matching for c and E. These measures aim to prevent HDFN. In the updated guideline four patients groups with a putative increased risk of alloimmunization were defined, on grounds of either underlying disease, transfusion frequency, or potential (hyper-)respondership. The four patient groups concern (1) patients with autoimmune hemolytic disease; (2) patients with myelodysplastic syndrome and (3) patients with an immediate early antibody (IEA) against a clinically relevant RBC antigen. For these three patient subgroups Rh phenotype (CcDEe) and K compatible RBCs are selected. Finally, the fourth group consists of patients with hemoglobinopathies (SCD or thalassemia) for whom Rh phenotype, K and Fy(a) compatible RBCs are selected, and whenever available, Jk(b), S or s compatible RBCs. The recommended matching strategies formulated in Dutch transfusion guidelines are summarized in Table 1 (16).

**Table 1:** Matching strategies for various patient groups as recommended in the 2011 Dutch Transfusion guideline.

**Patient group**	**Matching strategy**
Sickle cell anemia and thalassemia	Rh phenotype, K and Fy(a)
	(and if available, Jk(b), S and s)
Autoimmune hemolytic anemia	Rh phenotype and K
Myelodysplastic syndrome	Rh phenotype and K
Alloimmunized with clinical important antibodies	Rh phenotype and K
Woman of childbearing age	c, E and K

Apart from these specific patient groups, patients in the Netherlands are routinely tested for the presence of IEAs prior to RBC transfusions. When IEAs are detected, both their specificity and clinical importance are investigated. In case of a clinical important IEAs it is essential to select donor erythrocytes that are negative for corresponding antigens to prevent HTRs. Furthermore, dependent on the matching strategy, it may be required that donor erythrocytes are compatible with other antigens of the patient (extended matched), to prevent the formation of additional IEAs. Because antibodies may lose detectability over time, accurate recording and accessibility of patient antibody formation is of the utmost importance (17–19). Besides in-hospital records, a national database is available in the Netherlands (TRIX, Transfusion Register Irregular antibodies and X(cross)-matching), in which hospitals register patients with RBC antibodies and cross-match problems (20). This system is accessed for the evanesced antibodies in all patients with a transfusion request to prevent re-exposure to the cognate antigen. However, these registrations will not prevent re-exposure due to an inadequate antibody follow-up after transfusion.

The blood management model will have to accommodate matching strategies currently implemented as well as various extended matching strategies. The model should incorporate all costs involved for various matching strategies considered (e.g., costs of personnel and materials used).

## 4. Typing the donor population

Different matching strategies will pose different requirements on the availability of typed blood products. The required number of typed blood products, the variation in its demand, and the required service level (the probability of not being able to deliver a requested typed blood product) will determine the number of typed blood products that will have to be available in stock at any time, and hence the level of typed donors. A large typed donor population has the advantage that in most cases donor erythrocytes can be selected directly from inventory, even when blood products need to be typed negative for combinations of antigens. However, there will always be a balance between the additional efforts required to fulfill requirements for typed blood products and extending the pool of elaborately typed donors.

## 5. Donor recruitment

Transfusing matched blood is only feasible if there are enough donors that are typed negative for specific (combinations of) blood group antigens. For instance, many Blood Services in Western countries have a structural shortage of Fy(a)-neg, Fy(b)-neg, e-neg donors. This blood type is most common in populations from Sub-Saharan Africa, of which relatively few individuals are enrolled as blood donors (21). In addition, in many countries a broad variety of ethnic minority populations exist. Shifting immigration patterns and mixing of these populations will increase the demand for rare blood type combinations. A valuable side effect of recruiting among minority groups is a potentially increase of donors for HLA-matched substances of human origin, such as stem cells. Blood Services therefore need to identify which specific ethnic minority populations to focus on in terms of rare blood type prevalence.

## 6. Integration

**Figure 2:**
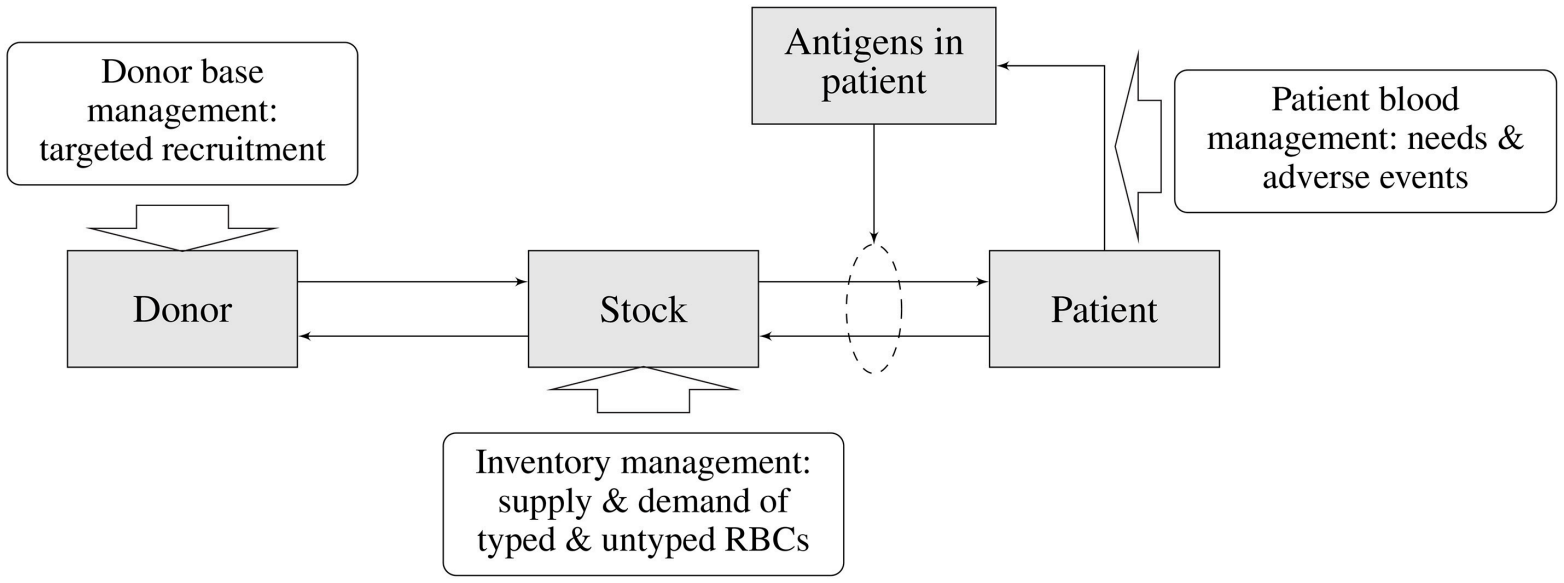
Main elements of the blood supply chain and associated sub-models of the blood management system.

In the previous sections various elements of the blood transfusion chain and their interdependencies were discussed (see Figure 1). Each of these elements and their interactions need to be modeled in order to allow evaluation of the impact of a particular matching strategy on the transfusion risk of patients (i.e., acute and delayed HTRs) and on other parts of the blood supply chain (e.g., the availability of matched blood products, costs of type and screen, storage, outdating, and targeted donor recruitment). The main elements of the blood supply chain and the associated sub-models describing various interactions required for an integrated blood management model is depicted in Figure 2.

The starting point for any evaluation is the blood matching strategy, as this, in combination with the patient mix, will determine the demand for particular blood products. Depending on the matching strategy and patient mix (patient subgroups) there will be a risk of antibody formation and subsequent risk for adverse transfusion complications. Moreover, the combination of patient mix and associated matching strategy will determine the demand for typed blood products in the inventory. The availability of typed blood products in the inventory is dependent on the availability of typed blood donors, which again is dependent on the efforts and requirements of targeted donor recruitment.

The assessment of the transfusion complication risk requires estimates of the likelihood of antibody formation and subsequent transfusion reactions in patients given a particular matching strategy. Such estimates should incorporate the transfusion pattern and the ethnic (blood type) composition of various patient sub-groups. Also, antigen specific estimates for the likelihood of developing antibodies as well as for transfusion complications are required. The likelihood of transfusion complications in combination with cost and the health impact will allow estimation and subsequent balancing of the costs and benefits from the matching strategy applied.

To enable matching blood for transfusion recipients antigen and antibody profiles of patient subgroups have to be determined. Next, compatible RBC units have to be selected from inventory. Detailed information on blood use and the antigen profiles per patient group allows assessment of the blood inventory required to meet patient needs. This will be a description of the required inventory both in terms of amount and composition of RBCs in various stocks along the blood transfusion chain. Blood product demand will show a stochastic behavior and a realistic blood management model will therefore have to be able to accommodate such random variations. Given the patient mix, matching strategy and associated transfusion characteristics, for any pre-specified acceptability rate for the unavailability of (matched) blood products and inventory management strategy, the required blood inventory size and composition can be determined. The resulting costs and effects for the complete blood transfusion chain (outdating, size of the inventory, logistics, and material handling costs) can now be estimated. Note that the unavailability of matched blood products will impact the likelihood of transfusion complications in patients. Therefore, optimization of the overall blood transfusion chain will require a separate sub-optimization for the inventory management strategy.

The availability of compatible RBC units required in the inventory is directly linked to the availability of typed donors and hence guides the typing strategy and targeted donor recruitment efforts. The typing strategy will be aiming at fulfilling the requirements for maintaining sufficient inventory levels, but this will be dependent on the availability of specific antigen profiles in the (typed) donor population. Whenever these are insufficient, targeted donor recruitment efforts will have to ensure adequacy of the desired antigen profiles in the un-typed donor population, and ultimately those in the typed donor population. Estimates for the costs of recruiting specific donor subgroups in order to ensure a sufficient level of typed blood groups in the donor population are required to estimate the costs for maintaining the required inventory levels. Other than in the inventory management, which is an in-line process, it is presumed that the required levels of typed donors will be met by increasing donor recruitment efforts.

## 7. Discussion

In this article we discussed a conceptual framework for a blood management model which allows optimization of blood matching strategies. The model links various elements from the blood transfusion chain to allow an assessment of the full impact of any particular matching strategy. The approach is unique in the sense that in the past matching strategies were guided by the prevention of transfusions complications observed with the administration of blood products, without consideration its impact on the underlying blood supply process. In theory this new approach seems sensible, however, in practice there will be a number of complicating factors.

First of all, except for some specific patient subgroups there is only limited evidence available on the effectiveness of matching strategies for the prevention of transfusion complications. Despite the fact that transfusion complications are accurately analyzed, patient exposure is far more difficult to ascertain. More evidence however has been gained for the risks of alloimmunization in various patient cohorts in the Netherlands in the ongoing Risk-Factors for alloimmunization after red blood Cell Transfusion (R-FACT) study (22). This concerted collaboration of several large hospitals will provide the information required to model risk factors for some patient subgroups. Also, looking back at the reduction of transfusion complications after implementation of altered matching strategies may support inference on its effectiveness. However, this effect may also be confounded by transfusion practice.

Another complicating factor is the impact of transfusion complications on patients, as this may vary from serologic observations or mild symptomatic anemia to life-threatening complications and death. Not only are predictors for predisposing factors lacking, but the impact of various levels of transfusion complications on patient health (apart from death) are not readily available, and neither are the associated costs. Assessing costs of complications is complex as it requires separation of the costs of patient treatment from costs of complications which are confounded by definition. Similar complications occur when estimating the impact on patient health. Nonetheless, an increasing number of publications on the impact of transfusion complications are becoming available (23–25).

In most settings detailed information on transfusion practice (number of transfused blood products for specific patient subgroups and the variation herein) is lacking. In the PROTON II study for a large number of Dutch hospitals detailed information on blood transfusions administered to patients is collected in one central datawarehouse (26). These data consist not only of transfused products, but also patient diagnosis and lab results. These data are indispensable when modeling the logistics of the blood supply in general, and for specific patient groups. Optimized inventory and dispatching strategies can be developed for both hospital and regional distribution centers and may be tailored to specified matching strategies. Note that with data on blood use the requirements and constraints for such models are available.

For the assessment of the risk of transfusion reactions (depending on the matching strategy) information on historical exposure of patients to blood products is required in order to assess the likelihood of antibody development. Such data is at present only available at a large scale for Denmark and Sweden where long term follow-up data on transfused patients is recorded in the SCANDAT database (27, 28). Such information may be used to estimate an approximate risk of exposure to red blood cells in other settings.

The development of an integrated blood management model will increase transparency in costs and effects of selected matching strategies and is therefore -if applied- expected to contribute to an improved efficiency in blood transfusion practice.

## Author contributions

JvS, PdW, JL, BV, KvdH, AvD, MK, MvK, CvdS, HS, WdK, and MJ design of the framework. JvS, PdW, BV, KvdH, AvD, HS, and MJ initial draft of the paper. All authors review and update of the final paper.

### Conflict of interest statement

The authors declare that the research was conducted in the absence of any commercial or financial relationships that could be construed as a potential conflict of interest. The reviewer CJ declared a past co-authorship with one of the authors WdK to the handling Editor.
